# Assessing physical abilities of sarcopenia patients using gait analysis and smart insole for development of digital biomarker

**DOI:** 10.1038/s41598-023-37794-7

**Published:** 2023-06-30

**Authors:** Shinjune Kim, Seongjin Park, Sangyeob Lee, Sung Hyo Seo, Hyeon Su Kim, Yonghan Cha, Jung-Taek Kim, Jin-Woo Kim, Yong-Chan Ha, Jun-Il Yoo

**Affiliations:** 1 Department of Biomedical Research Institute, Inha University Hospital, Incheon, Republic of Korea; 2 Department of Biomedical Research Institute, Gyeongsang National University Hospital, Jinju, Republic of Korea; 3 Department of Orthopaedic Surgery, Daejeon Eulji Medical Center, Daejeon, Republic of Korea; 4 Department of Orthopedic Surgery, Ajou University School of Medicine, Suwon, Republic of Korea; 5 Department of Orthopaedic Surgery, Nowon Eulji Medical Center, Seoul, Republic of Korea; 6 Department of Orthopaedic Surgery, Bumin Medical Center, Seoul, Republic of Korea; 7 Department of Orthopedic Surgery, Inha University Hospital, 27, Inhang-ro, Jung-gu, Incheon, Republic of Korea

**Keywords:** Biotechnology, Biomarkers, Medical research

## Abstract

The aim of this study is to compare variable importance across multiple measurement tools, and to use smart insole and artificial intelligence (AI) gait analysis to create variables that can evaluate the physical abilities of sarcopenia patients. By analyzing and comparing sarcopenia patients with non sarcopenia patients, this study aims to develop predictive and classification models for sarcopenia and discover digital biomarkers. The researchers used smart insole equipment to collect plantar pressure data from 83 patients, and a smart phone to collect video data for pose estimation. A Mann–Whitney U was conducted to compare the sarcopenia group of 23 patients and the control group of 60 patients. Smart insole and pose estimation were used to compare the physical abilities of sarcopenia patients with a control group. Analysis of joint point variables showed significant differences in 12 out of 15 variables, but not in knee mean, ankle range, and hip range. These findings suggest that digital biomarkers can be used to differentiate sarcopenia patients from the normal population with improved accuracy. This study compared musculoskeletal disorder patients to sarcopenia patients using smart insole and pose estimation. Multiple measurement methods are important for accurate sarcopenia diagnosis and digital technology has potential for improving diagnosis and treatment.

Sarcopenia is an age-related decrease in muscle mass, strength, and function. It is a common problem among older people and can lead to reduced mobility, increased risk of falls, fractures and reduced quality of life^1^. The causes of sarcopenia are complex, including hormonal changes, reduced physical activity, oxidative stress and inflammation, and changes in muscle protein synthesis and breakdown^2,3^. Several guidelines have been developed to diagnose sarcopenia, and there are representative guidelines presented by institutions such as EWGSOP and AWGS^4–6^. These diagnostic guidelines include physical function evaluation items for patients with sarcopenia, which are currently being measured in various ways^1,7,8^.

Diagnosing sarcopenia involves assessing muscle mass, strength, physical performance, and body composition through various methods. A recent focus has been on evaluating physical performance, with tools like the Gait Speed Test, Chair Stand Test, Timed Up and Go (TUG) Test, and Handgrip Strength Test being commonly used^9,10^. However, these methods are susceptible to subjective bias from the measurer or the environment. To address this, there has been a push towards using artificial intelligence (AI) to gather physical performance data^11^. In particular, studies such as calculating joint angles and ranges using pose estimation are being actively discussed^12,13^.

Research is underway to enhance the measurement accuracy of patients' physical performance using smart equipment, alongside AI technologies such as body pose estimation^14–16^. Pose estimation is a computer vision technology that uses deep learning models to estimate human body key points in real-time. It tracks and detects human body joints and parts, allowing for 2D or 3D pose estimation^17–19^. Currently, there is active research being conducted to compare its accuracy and usefulness with VICON motion system (Vicon Nexus; Vicon Motion Systems Ltd., Oxford, England), which uses multiple cameras to perform highly accurate 3D motion capture^12,20^. Through these comparative studies, the accuracy and usefulness of the pose estimation method is being verified^12,20,21^. In addition, various wearable devices such as smartwatches and smart insoles are currently being used to measure patients' physical performance. In particular, research utilizing Inertial Measurement Unit (IMU) sensors such as the Smart Insole is actively being conducted in the field of muscular dystrophy, and significant spatial and temporal parameters are being identified. As an example, there have been studies analyzing the gait of osteoporosis and muscular dystrophy using AI and wearable sensors. There have also been studies identifying patients with muscular dystrophy using IMU sensors^22,23^.

In current research on various musculoskeletal patients, including sarcopenia, it is common to use a single measurement tool for analysis. However, this approach may not fully capture the diversity of variables that each tool can measure, and may not accurately reflect the relative importance of variables when comparing across tools. Therefore, to develop predictive and classification models for sarcopenia and discover digital biomarkers, it is crucial to compare variable importance across multiple measurement tools and find a simple, accurate assessment tool. The purpose of this study is to use smart insole and AI gait analysis together to create variables that can evaluate the physical abilities of sarcopenia patients, before expanding to predictive and classification models, and to compare and analyze sarcopenia patients with healthy individuals.

## Materials and methods

### Subjects

In order to collect insole and pose esimation data for sarcopenia, GNUH (Gyeongsang National University Hospital, Jinju, South Korea) conducted a study on 83 patients with musculoskeletal disorders in 2022. Of the 83 patients with musculoskeletal disorders, 23 were pre-judged to have sarcopenia. Among the 23 sarcopenia patients, there were 15 females and 8 males, while the Control group consisted of 23 and 31 individuals, respectively (refer to Supplementary Table S1). The study adhered to the principles of the Declaration of Helsinki and was approved by the IRB at Gyeongsang National University Hospital. All research procedures were carried out with strict adherence to ethical standards, including protection of participants' privacy, confidentiality, and rights.

**Figure 1:**
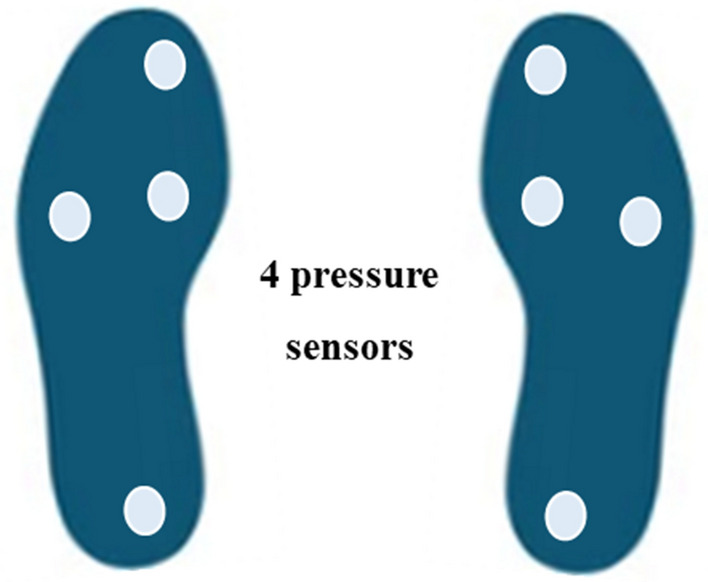
Smart Insole for gait analysis.

**Figure 2:**
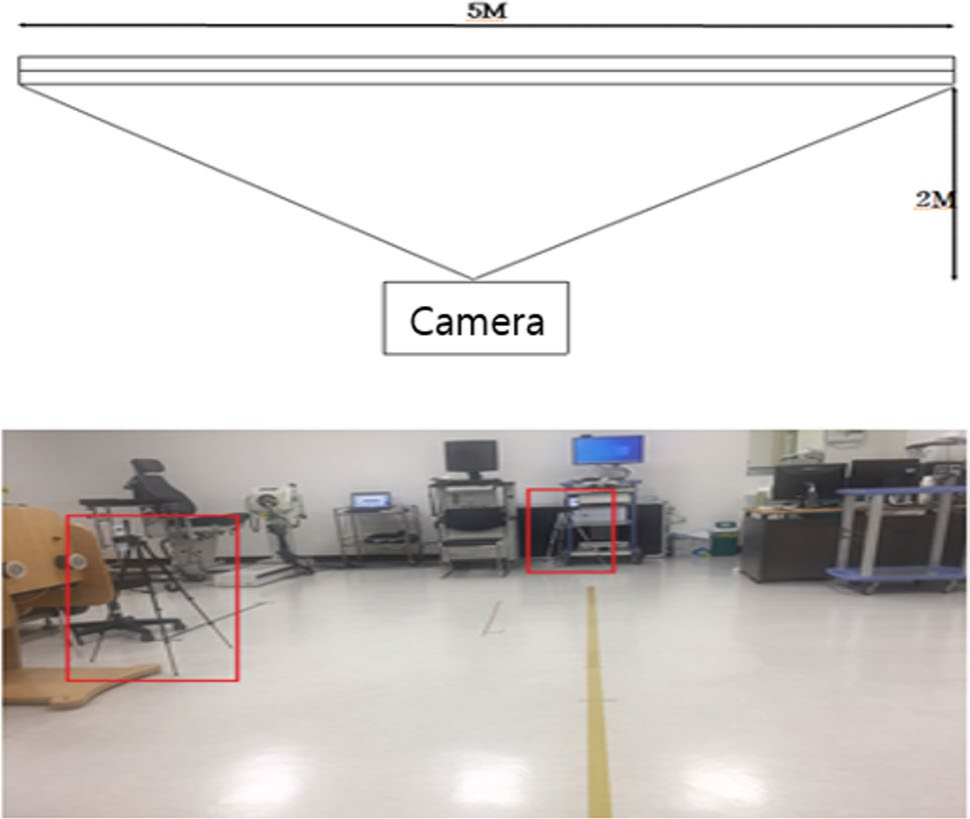
Video recording protocol.

To collect data from the insoles of 83 patients, we used the Smart Insole equipment from SALTED (Seoul, South Korea), which is equipped with four pressure sensors and three-axis IMU sensors for each insole, as shown in Fig. 1^24^. The insoles wirelessly transmitted four-channel foot pressure and three-channel acceleration data at a sampling rate of 30 Hz. To collect plantar pressure data, each patient wore the insoles and walked for one minute^16^. A smart phone (Galaxy A20, Samsung Electronics) equipment was used to collect video data to be used for pose estimation. The measurement was conducted using the rear camera of a smartphone, and the recorded video had a resolution of 1080p and a frame rate of 30fps. As for the video recording protocol, as shown in Fig. 2, a lateral walking video was recorded once for a walking distance of 5 m. In addition, the distance between the patient and the camera was based on a vertical distance of 2 m and a height of 1.3 m from the floor. There are no standardized measurement distances and heights. For this study, a measurement distance of 5 m was used and the minimum distance of 2 m was set to fit the entire screen. For the height from the ground, we used approximately 1.3, the height of a person's shoulders, for angle and horizontal correction.

### Analysis methods

**Figure 3:**
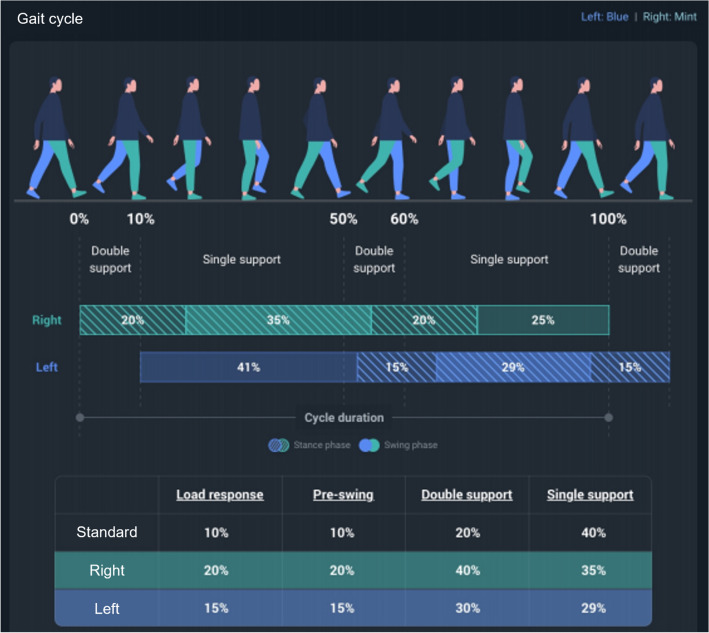
Gait cycle captured by smart inasole.

We used the SALTED Smart Insole (Seoul, South Korea) equipment to collect plantar pressure data from 83 patients. The insoles, as shown in Fig. 1, were equipped with four pressure sensors and one three-axis IMU sensor for calibration purposes. Each patient wore the insoles while walking for one minute, and the pressure data were collected using the four pressure sensors. The SALTED internal program was then utilized to calculate relevant variables as shown in Fig. 3.

**Figure 4:**
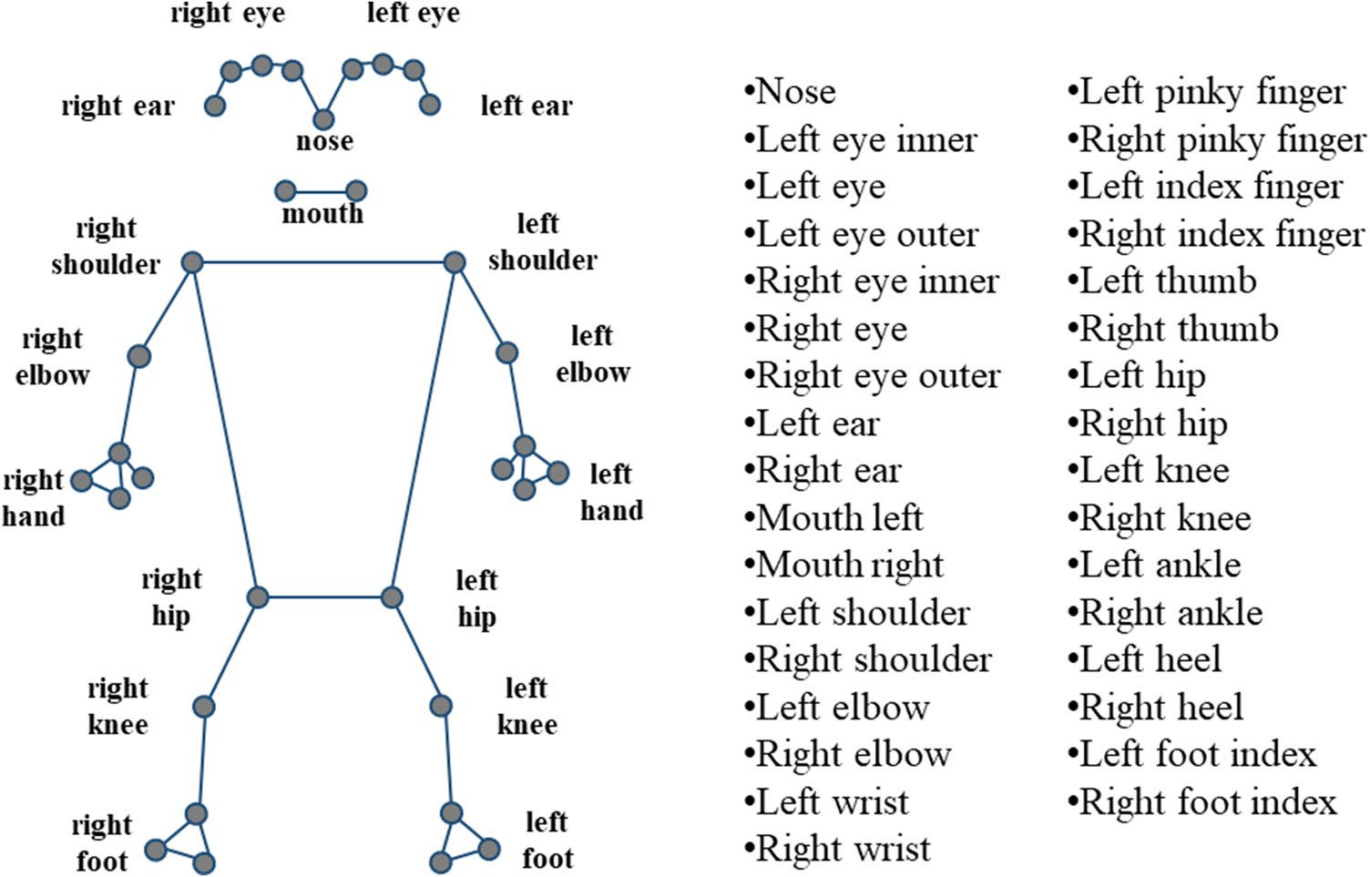
Visualization of Blaze Pose’s 33 key points in human pose estimation.

To estimate and analyze the patient's pose, we employed video analysis for pose estimation using the Dr.log application and the DMS system (Deevo, Jinju, Korea)^25,26^. The Dr.log application collected patients' gait videos in real-time and stored them in a database. The DMS system performed real-time pose estimation from the collected database. For the pose estimation process, we utilized Mediapipe, an open-source software developed by Google that uses a Convolutional Neural Network (CNN) model based on neural network-based deep learning algorithms. The CNN model includes a convolutional layer and a pooling layer to extract features from the input image, followed by fully connected and softmax layers. Using Mediapipe's pose estimation function, we estimated a total of 33 key points, with 25 representing the upper body and 8 representing the lower body, utilizing the Blaze Pose model, as shown in Fig. 4^27^.

We utilized R Studio, a statistical analysis program, to analyze the centrality and variability of all collected patient data. In addition, due to the small sample size, we conducted Shapiro normality tests and used Mann–Whitney U tests when normality assumptions were not met. We compared the sarcopenia group and control group using R Studio and created image visualizations for each group's results. The significance level was set at *p < 0.1, **p < 0.01, and ***p < 0.001.

### Ethical standards

The study adhered to the principles of the Declaration of Helsinki and was approved by the IRB at Gyeongsang National University Hospital. (IRB No. GNUH 2022-01-032-008) All research procedures were carried out with strict adherence to ethical standards, including protection of participants’ privacy, confidentiality, and rights.

## Results

We conducted a normality test on each variable in both the sarcopenia group (n = 23) and control group (n = 60). The test indicated that the majority of variables in the sarcopenia group did not adhere to the normality assumption (refer to Supplementary Table S2). Therefore, we compared the variables between the two groups using the Mann–Whitney U test. The measurement values are presented in Tables 1 and 2, and the *p*-values for each variable are shown at the bottom of each table. Table 1 presents the characteristics of the two groups using smart insole, while Table 2 presents the characteristics using pose estimation method (refer to Supplementary Figs. S1 and S2). The smart insole provides a total of 6 variables (Total number of steps, Cadence, R double support, R single support, L double support, L single support) and similarly, the pose estimation provides results for 23 variables representing each joint point. The *p*-values are provided for all 6 variables in the case of the smart insole, and only for 15 variables excluding the maximum and minimum values in the case of pose estimation. The description of the 23 variables provided by pose estimation is presented in Supplementary Table S3. In order to assess the detection accuracy of pose estimation using Mediapipe, key points representing the head, shoulders, elbows, wrists, hips, knees, and ankles were utilized as reference points. The detection criterion was based on cases where the marker positions were flipped or estimation was not performed. A total of 100 images were evaluated for this purpose. The accuracy was determined by considering whether the estimation was successful for each of the 15 keypoints. The resulting detection accuracy for the 15 keypoints was measured at 89.23%.

**Table 1:** Characteristics of sarcopenia group and control group measured using smart insole.

	Total number of steps (n)	Cadence (steps/min)	R_double_support (%)	R_single_support (%)	L_double_support (%)	L_single_support (%)	Observations
Sarcopenia							
Mean	84.32	87.42	17.44	36.30	17.00	36.97	23
Median	88.00	89.00	17.35	35.81	17.00	37.34	
Standard deviation	22.99	22.28	3.77	5.97	4.31	7.09	
Range	87.00	79.00	13.36	24.91	17.10	28.54	
Min	31.00	39.00	11.34	25.03	7.60	19.69	
Max	118.00	118.00	24.70	49.94	24.70	48.23	
Control							
Mean	88.32	88.04	17.08	36.74	17.22	37.22	60
Median	87.50	91.50	18.97	38.48	19.31	37.58	
Standard deviation	24.49	17.24	4.98	6.90	4.86	5.55	
Range	115.00	68.00	19.92	30.36	17.89	25.79	
Min	36.00	48.00	5.05	19.60	6.40	20.39	
Max	151.00	116.00	24.97	49.96	24.29	46.18	

Mann–Whitney U test	Total number of steps (n)	Cadence (steps/min)	R_double_support (%)	R_single_support (%)	L_double_support (%)	L_single_support (%)	
*p*-value	0.841	0.756	0.798	0.388	0.563	0.868	

*p-value < 0.1, **p-value < 0.01, ***p-value < 0.001.

**Table 2:** Characteristics of sarcopenia group and control group measured using pose estimation.

	Knee_mean	Knee_max	Knee_min	Knee_range	Hip_mean	Hip_max	Hip_min	hip_range
Sarcopenia								
Mean	165.81	179.78	135.14	42.97	4.49	16.01	0.04	16.09
Median	165.48	179.90	135.84	43.08	4.24	16.06	0.03	16.04
Standard deviation	2.25	0.23	2.74	3.78	0.66	1.84	0.02	1.85
Range	9.50	0.80	9.19	19.64	2.81	8.82	0.10	8.81
Min	162.15	179.19	129.80	29.48	2.90	11.58	0.01	11.55
Max	171.64	179.98	138.98	49.11	5.71	20.40	0.11	20.36

	Shoulder_angle_mean	Shoulder_angle_max	Shoulder_angle_min	Shoulder_angle_range	Ankle_mean	Ankle_max	Ankle_min	Ankle_range
Sarcopenia								
Mean	148.29	178.81	91.63	87.26	126.40	148.17	103.95	44.61
Median	147.60	179.81	90.81	88.20	125.90	148.49	103.08	45.40
Standard deviation	7.82	2.58	1.66	2.57	2.61	1.56	2.95	3.03
Range	29.06	11.46	5.91	11.17	10.68	5.66	12.18	11.28
Min	131.41	168.54	90.01	78.52	121.79	144.15	100.63	37.44
Max	160.47	180.00	95.92	89.69	132.47	149.81	112.81	48.72

	All_max_dif	Hipknee_dif	Hipankle_dif	Kneeankle_dif	Knee_dif	Ankle_dif	Hip_dif	Observations
Sarcopenia								
Mean	44.06	7.24	52.11	42.06	0.06	47.25	15.70	23
Median	43.04	7.28	51.45	39.68	0.05	45.09	16.05	
Standard deviation	7.07	1.03	6.27	8.90	0.05	7.47	1.83	
Range	28.11	4.86	27.47	32.26	0.19	28.96	8.82	
Min	35.78	4.93	40.33	30.77	0.00	37.43	11.58	
Max	63.89	9.79	67.80	63.03	0.19	66.39	20.40	

	Knee_mean	Knee_max	Knee_min	Knee_range	Hip_mean	Hip_max	Hip_min	Hip_range
Control								
Mean	165.33	179.46	137.11	44.02	5.72	18.30	0.06	16.10
Median	165.32	179.41	135.51	45.51	5.35	17.21	0.05	16.55
Standard deviation	2.27	0.30	4.37	4.61	1.31	2.91	0.05	1.87
Range	10.51	1.75	19.70	19.39	4.88	14.75	0.19	11.18
Min	161.13	178.21	130.74	29.48	4.11	11.58	0.00	11.06
Max	171.64	179.96	150.43	48.87	8.99	26.32	0.20	22.24

	Shoulder_angle_mean	Shoulder_angle_max	Shoulder_angle_min	Shoulder_angle_range	Ankle_mean	Ankle_max	Ankle_min	Ankle_range
Control								
Mean	154.05	179.48	91.29	89.85	123.03	146.52	102.69	45.76
Median	153.66	179.58	91.46	90.21	122.97	146.52	102.43	46.13
Standard deviation	3.90	0.65	1.50	2.22	1.87	1.86	1.77	2.19
Range	28.16	4.88	9.12	10.99	9.50	8.33	7.77	13.98
Min	131.41	175.11	86.28	84.52	120.11	141.52	100.11	41.15
Max	159.58	179.99	95.40	95.51	129.61	149.85	107.88	55.13

	All_max_dif	Hipknee_dif	Hipankle_dif	Kneeankle_dif	Knee_dif	Ankle_dif	Hip_dif	Observations
Control								
Mean	52.61	7.97	62.69	52.91	0.10	62.84	36.51	60
Median	52.05	7.80	63.09	50.43	0.09	63.14	35.34	
Standard deviation	6.16	0.99	4.12	6.56	0.05	5.11	8.35	
Range	30.64	4.03	21.11	26.98	0.26	24.87	35.86	
Min	36.00	6.14	51.12	42.46	0.02	47.68	21.58	
Max	66.64	10.16	72.24	69.44	0.28	72.55	57.43	

Mann–Whitney U test	Knee_mean	Knee_range	Hip_mean	Hip_range	Shoulder_angle_mean	Shoulder_angle_range	Ankle_mean	Ankle_range
*p*-value	0.433	< 0.001***	< 0.001***	0.294	0.002**	< 0.001***	< 0.001***	0.252

Mann–Whitney U test	All_max_dif	Hipknee_dif	Hipankle_dif	Kneeankle_dif	Knee_dif	Ankle_dif	Hip_dif	
*p*-value	< 0.001***	0.003**	< 0.001***	< 0.001***	< 0.001***	< 0.001***	< 0.001***	

*p-value < 0.1, **p-value < 0.01, ***p-value < 0.001.

The characteristics of the Sarcopenia and Control groups were investigated using smart insole technology, and the results are summarized in Table 1. The Sarcopenia group had a mean total number of steps of 84.32 steps and a cadence of 87.42 steps/min. For the R double support, R single support, L double support, and L single support means, the values were 17.44%, 36.30%, 17.00%, and 36.97%, respectively. In comparison, the Control group had a mean total number of steps of 88.32 steps and a cadence of 88.04 steps/min. The means for R double support, R single support, L double support, and L single support were 17.08%, 36.74%, 17.22%, and 37.22%, respectively. The Mann–Whitney U test revealed no significant differences between the two groups for any of the variables. However, caution should be taken when interpreting the results, as the high *p*-values for the total number of steps, cadence, R double support, R single support, L double support, and L single support suggest that small sample sizes may have influenced the findings.

The Dr.log site was used to extract joint point data from the image through pose estimation, and Dr.log DMS was then employed to identify time series patterns and characteristic values for coordinate information. The Mann–Whitney U test results classified the 15 variables studied into three groups based on their *p*-values. The first group, including knee mean (*p* = 0.433), ankle range (*p* = 0.252), and hip range (*p* = 0.294), had *p*-values greater than 0.1, indicating that these variables did not exhibit a significant difference between the two groups. The second group, consisting of hip mean, shoulder angle range, ankle mean, all max dif, hipankle dif, kneeankle dif, knee dif, ankle dif, and hip dif, had *p*-values less than 0.001, demonstrating a significant difference between the two groups. The third group, composed of hipknee dif and shoulder angle mean, had *p*-values of 0.003 and 0.002, respectively, indicating that they also displayed a significant difference between the two groups, although to a lesser extent than the second group. In summary, the results suggest that using pose estimation reveals a significant difference in joint angle measurements between the two groups, which could be useful in understanding the fundamental cause of movement pattern differences between them.

## Discussion

There are different approaches to measure gait, with smart insoles and pose estimation being two commonly used methods. Smart insole is a device that can be inserted into a shoe to measure various parameters of the foot during gait, such as pressure distribution, force, and acceleration^28,29^. The device contains sensors that collect data, which is then sent to a computer for analysis. The benefits of using smart insoles include detailed information about the foot's biomechanics, ease of use, non-invasiveness, and no need for special setup^29,30^. In contrast, pose estimation is a computer vision technique that uses a camera to track joint movement in the body^15,31^. By recording a person's movements as they walk or run, the software can estimate the position of joints in the body. The advantage of pose estimation is that it provides a comprehensive view of the entire body, including the limbs, spine, and pelvis, making it easier to study external variables such as gait asymmetry^32^. However, it can be more challenging to set up and requires more technical expertise.

There were significant differences between the two methods in this study. Smart insole primarily focuses on the foot, providing detailed information on foot biomechanics^33^. Specifically, there are areas that pose estimation fails to detect, such as ground reaction forces on both feet, and it has advantages in calculating variables like single support and double support. It is more practical to use in clinical settings, as space limitations do not occur. However, smart insole may not provide a complete picture of the body's movement during gait, leading to large deviations in the measured variables. Furthermore, in musculoskeletal patients, it is crucial to verify whether consistent time series patterns are present even when sufficient foot pressure is not applied. Pose estimation provides a more comprehensive view of the entire body, detecting asymmetries and compensations in gait^18,31,32^. The study showed significant differences in most variables using pose estimation, particularly the angle of maximum opening in the shoulder, ankle, and stationary posture. However, there was also a limitation that measuring with pose estimation required sufficient space and proper filming location.

Patients with sarcopenia exhibit gait changes due to the progressive decline in skeletal muscle mass, strength, and function. These changes are characterized by decreased single support time and increased double support time, primarily resulting from reduced muscle strength and impaired balance^34,35^. However, in our study, the smart insole was unable to fully capture these distinctive gait characteristics. In contrast, pose estimation accurately represented the gait cycle characteristics, as demonstrated in Tables 1 and 2. Specifically, the smart insole showed difficulty in identifying significant differences between the elderly patient group and the control group of relatively young and healthy individuals. On the other hand, pose estimation offered the advantage of estimating markers that could capture a wider range of functional variables. Compared to smart insole, pose estimation allowed for the consideration of a greater number of biomarkers related to various body functions. Therefore, using pose estimation for comparing the sarcopenia group with the non-sarcopenia group provided a broader perspective in assessing gait characteristics.

Research on sarcopenia is a rapidly evolving field, with ongoing efforts to identify digital biomarkers using various approaches. While previous studies have focused on using a single measurement device to identify biomarkers, it is becoming increasingly clear that a more comprehensive approach is needed. This requires the integration of multiple devices and variables to account for the complex nature of the disease. Therefore, further research is needed to combine the variables from existing smart insole and pose estimation studies to develop a predictive model and identify novel digital biomarkers.

## Limitations

When conducting pose estimation for muscle function evaluation in sarcopenia, the following limitations exist: (1) Pose detection accuracy: lighting, camera angle, and clothing can limit the accuracy of pose estimation for sarcopenia diagnosis; (2) Environmental constraints: environmental factors such as background clutter and movement can hinder pose estimation's ability to assess muscle function. Similarly, with the case of smart insoles, the following limitations exist: (1) Inaccuracy of sensors: The accuracy of smart insole’s IMU sensors can be affected due to the limitation of having only one IMU sensor for calibration purposes. Sensor drift, which refers to the gradual deviation of the sensor readings over time, can occur and cause inaccuracies in the collected data, which can affect the accuracy of the plantar pressure data results.; (2) Environmental constraints: Environmental conditions like uneven or slippery surfaces can impact the ability of smart insoles to evaluate muscle function.

Additionally, both methods have the following common limitations: (1) User dependence: the results of muscle function evaluation using smart insoles and pose estimation can be affected by factors such as correct wearing of insoles and the patient's physical characteristics; (2) Limitations in capturing physical parameters: using smart insoles and pose estimation for muscle function evaluation can result in inaccuracies if all the important physical parameters like muscle tension and adjustment are not included; (3) Lack of standardized protocol: concerns about the reliability of results can arise due to the absence of a standardized protocol for the use of smart insoles in muscle function evaluation and the absence of standardized camera equipment and shooting method protocols in the case of pose estimation.

Lastly, in this study, a comparison was conducted between 23 sarcopenic patients and 60 individuals without sarcopenia. During the process of comparing the groups, characteristics such as gender and age were not matched due to the limitation of a small sample size. To address this, the analysis results of the groups with matched characteristics are provided in Supplementary Table S4. In this case, when comparing the results with the original analysis in Tables 1 and 2, no significant differences in variables were observed.

## Conclusion

In this study, a control group of 60 individuals with musculoskeletal disorders was compared to a group of 23 individuals with sarcopenia using both smart insole and pose estimation. The results indicated that the smart insole did not show any significant differences between the two groups, whereas the pose estimation variables showed significant differences in 12 out of 15 variables. These findings highlight the importance of using multiple measurement methods to develop accurate models for predicting and classifying sarcopenia. With the recent advancements in measurement technology, the accuracy of sarcopenia diagnosis has improved, and it is expected that more digital biomarkers will be discovered and utilized in future treatments. This underscores the potential of utilizing digital technology to improve the diagnosis and treatment of musculoskeletal disorders and sarcopenia.

## Supplementary Information

Supplementary Figures.

Supplementary Tables.

## Data Availability

The data used in this study were collected at Gyeongsang National University Hospital, and inquiries about the data should be directed to the author J.I.Y.
