# Supplementary material for: Assessing physical abilities of sarcopenia patients using gait analysis and smart insole for development of digital biomarker
Source: Sci Rep. 2023 Jun 30;13:10602. doi: 10.1038/s41598-023-37794-7 (PMC10313812; doi:10.1038/s41598-023-37794-7)
Supplement: Supplementary file 1 — Supplementary Figures. [file 41598_2023_37794_MOESM1_ESM.pptx]

## Slide 1
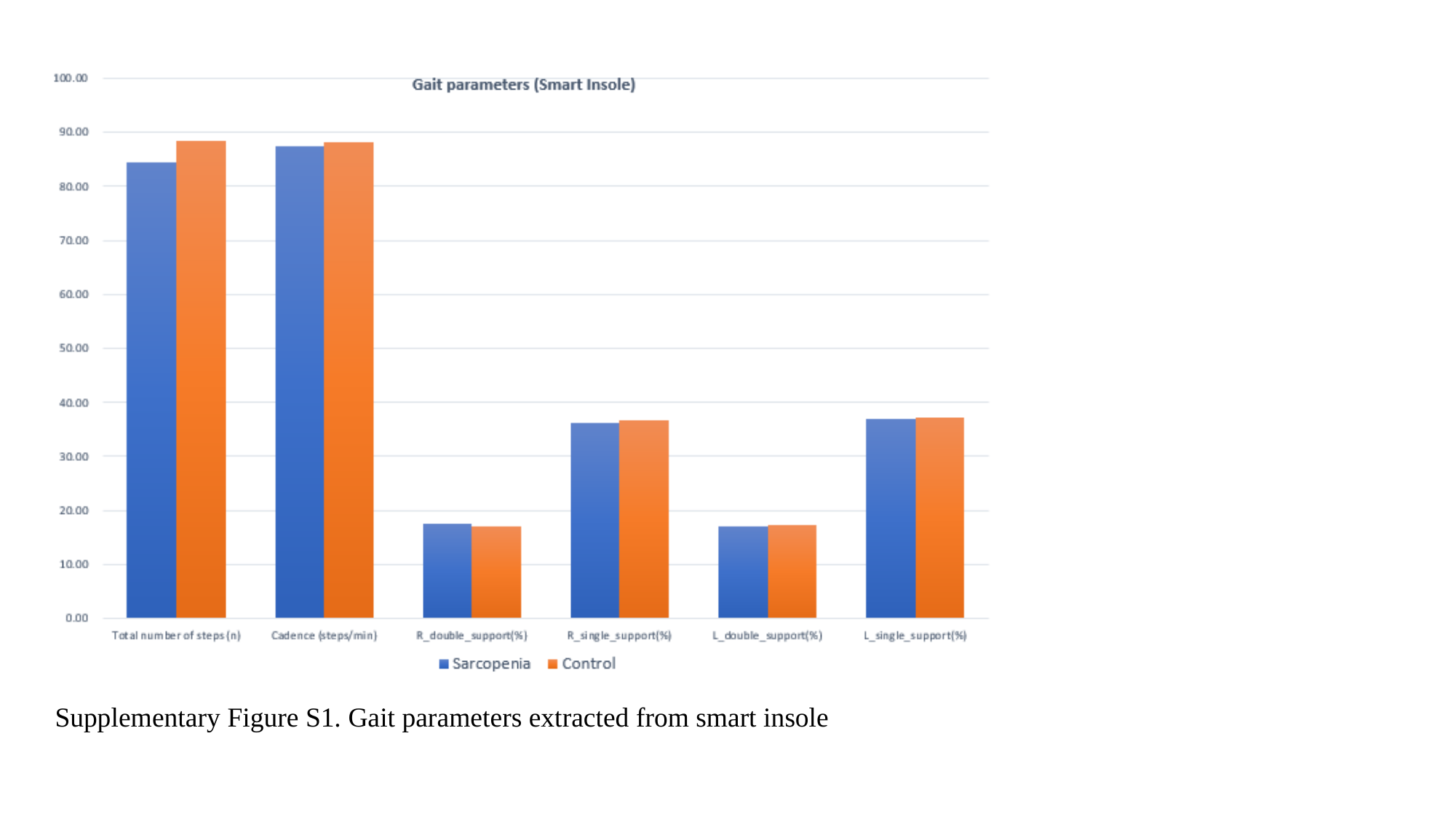

Supplementary Figure S1. Gait parameters extracted from smart insole

## Slide 2
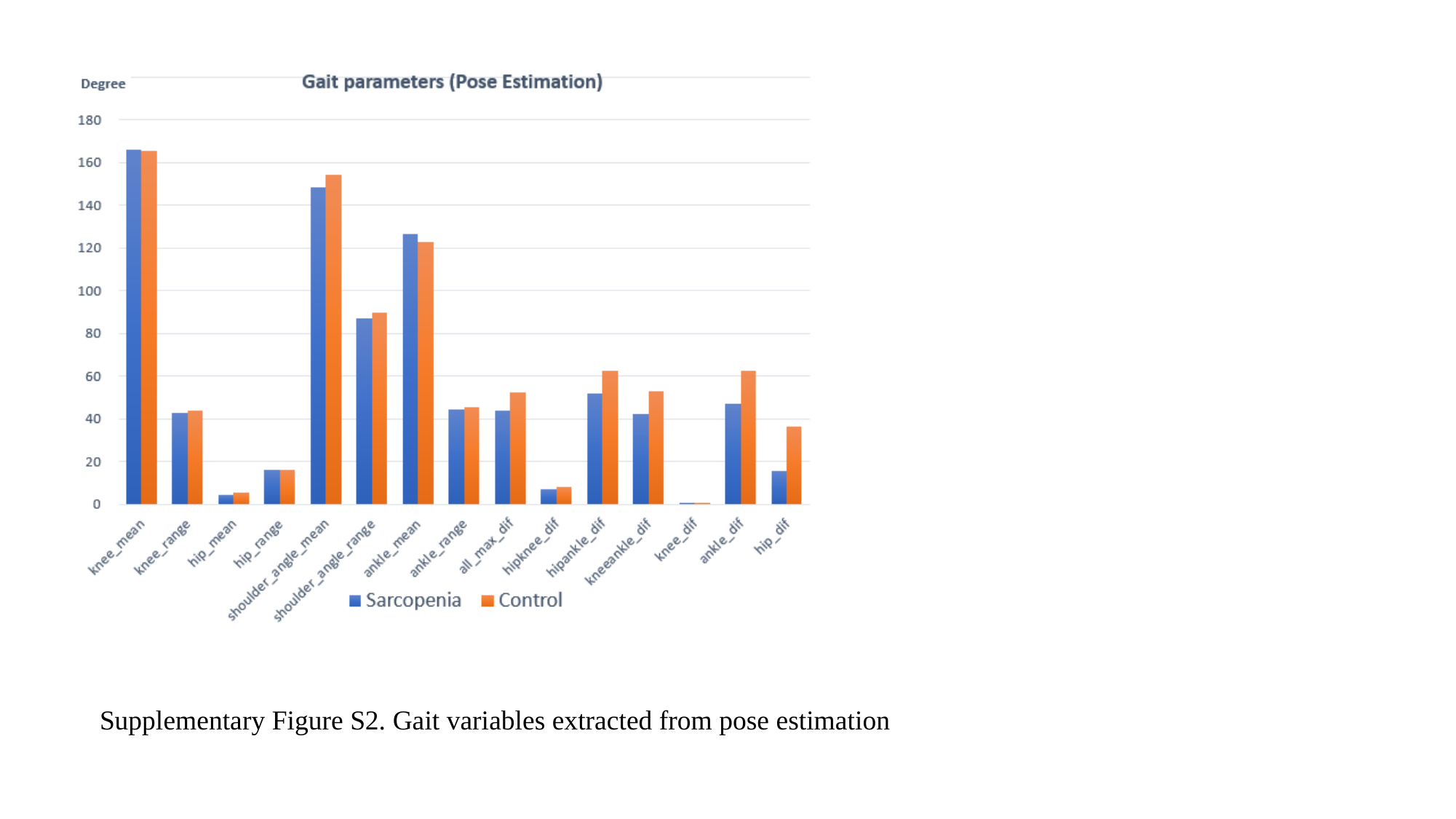

Supplementary Figure S2. Gait variables extracted from pose estimation
