# Supplementary material for: Assessing physical abilities of sarcopenia patients using gait analysis and smart insole for development of digital biomarker
Source: Sci Rep. 2023 Jun 30;13:10602. doi: 10.1038/s41598-023-37794-7 (PMC10313812; doi:10.1038/s41598-023-37794-7)
Supplement: Supplementary file 2 — Supplementary Tables. [file 41598_2023_37794_MOESM2_ESM.docx]

**Supplementary Table S1.** Characteristics of Sarcopenia and Control Groups

|  | | **Sex** | **Age (sd)** | Height **(sd)** | **Weight (sd)** |
| --- | --- | --- | --- | --- | --- |
| **Sarcopenia** | **Female** | **15** | 77.5(9.3) | 153.0(5.6) | 50.1(7.2) |
|  | **Male** | 8 | 78.4(7.5) | 168.2(8.3) | 63.5(8.1) |
|  | **Total** | 23 | 77.8(8.6) | 158.3(9.9) | 54.8(9.8) |
|  | | **Sex** | **Age (sd)** | Height **(sd)** | **Weight (sd)** |
| **Control** | **Female** | **29** | 69.0(16.5) | 155.4(5.9) | 55.2(10.8) |
|  | **Male** | 31 | 58.1(12.4) | 169.5(5.6) | 71.7(9.9) |
|  | **Total** | 60 | 63.4(15.4) | 162.7(9.1) | 63.7(13.2) |

(Sex : n, Age : years, Height : cm, Weight : kg)

**Supplementary Table S2.** Shapiro-Test Results for Normality Testing of Control and Sarcopenia Groups

| **Methods** | **Variables** | **Statistic (Control)** | **P-value(Control)** | **Statistic (Sarcopenia)** | **P-value(Sarcopenia)** |
| --- | --- | --- | --- | --- | --- |
| **Smart Insole** | **Left_double_support** | **0.884** | **0.001**^**^ | **0.980** | **0.960** |
|  | **Left_single_support** | **0.938** | **0.007**^**^ | **0.967** | **0.731** |
|  | **Right_double_support** | **0.909** | **0.003**^**^ | **0.979** | **0.959** |
|  | **Right_single_support** | **0.929** | **0.004**^**^ | **0.976** | **0.916** |
|  | **Total_walk** | **0.992** | **0.976** | **0.946** | **0.336** |
|  | **Cadence** | **0.960** | **0.058**^*^ | **0.944** | **0.314** |
| **Open pose estimation** | **Knee_meen** | **0.972** | **0.176** | **0.960** | **0.472** |
|  | **Knee_max** | **0.884** | **<0.001**^***^ | **0.757** | **<0.001**^***^ |
|  | **Knee_min** | **0.733** | **<0.001**^***^ | **0.936** | **0.144** |
|  | **Knee_range** | **0.735** | **<0.001**^***^ | **0.824** | **0.001**^**^ |
|  | **Hip_mean** | **0.905** | **<0.001**^***^ | **0.923** | **0.078**^*^ |
|  | **Hip_max** | **0.940** | **0.005**^**^ | **0.973** | **0.754** |
|  | **Hip_min** | **0.843** | **<0.001**^***^ | **0.872** | **0.007**^**^ |
|  | **Hip_range** | **0.770** | **<0.001**^***^ | **0.966** | **0.584** |
|  | **Shoulder_angle_mean** | **0.708** | **<0.001**^***^ | **0.966** | **0.585** |
|  | **Shoulder_angle_max** | **0.523** | **<0.001**^***^ | **0.518** | **<0.001**^***^ |
|  | **Shoulder_angle_min** | **0.939** | **0.005**^***^ | **0.850** | **0.003**^**^ |
|  | **Shoulder_angle_range** | **0.968** | **0.111** | **0.813** | **0.001**^**^ |
|  | **Ankle_mean** | **0.911** | **<0.001**^***^ | **0.970** | **0.683** |
|  | **Ankle_max** | **0.950** | **0.015**^*^ | **0.874** | **0.008**^**^ |
|  | **Ankle_min** | **0.938** | **0.004**^**^ | **0.834** | **0.001**^**^ |
|  | **Ankle_range** | **0.879** | **<0.001**^***^ | **0.892** | **0.017**^*^ |
|  | **All_max_dif** | **0.978** | **0.337** | **0.919** | **0.063**^*^ |
|  | **Hipknee_dif** | **0.939** | **0.005**^**^ | **0.943** | **0.213** |
|  | **Hipankle_dif** | **0.940** | **0.005**^**^ | **0.968** | **0.634** |
|  | **Kneeankgle_dif** | **0.913** | **<0.001**^***^ | **0.875** | **0.008**^**^ |
|  | **Knee_dif** | **0.908** | **<0.001**^***^ | **0.850** | **0.003**^**^ |
|  | **Ankgle_dif** | **0.940** | **0.005**^**^ | **0.900** | **0.026**^*^ |
|  | **Hip_dif** | **0.950** | **0.015**^*^ | **0.968** | **0.651** |

(^***^ : p-value<0.001, ^**^ : p-value<0.01, ^*^ : p-value<0.1)

**Supplementary Table S3.** Variables for joints used in gait analysis

| **Joint** | **Variables** | **Variables** |
| --- | --- | --- |
| **Shoulder** | **shoulder_range** | Shoulder angle range (from maximum to minimum) |
|  | **shoulder_min** | Minimum shoulder angle |
|  | **shoulder_max** | Maximum shoulder angle |
|  | **shoulder_mean** | Average shoulder angle |
| **Hip** | **hip_range** | Hip joint angular range (from maximum to minimum) |
|  | **hip_min** | Minimum hip joint angle |
|  | **hip_max** | Maximum hip joint angle |
|  | **hip_mean** | Average hip joint angle |
| **Knee** | **knee_range** | Knee angle range (from maximum to minimum) |
|  | **knee_min** | Minimum knee angle |
|  | **knee_max** | Maximum knee angle |
|  | **knee_mean** | Average knee angle |
| **Ankle** | **ankle_range** | Ankle angle range (from maximum to minimum) |
|  | **ankle_min** | Minimum ankle angle |
|  | **ankle_max** | Maximum ankle angle |
|  | **ankle_mean** | Average ankle angle |
| **else** | **hip_dif** | The difference between the angle while standing before walking and the maximum angle during walking (hip joint). |
|  | **ankle_dif** | The difference between the angle while standing before walking and the maximum angle during walking (ankle). |
|  | **knee_dif** | The difference between the angle while standing before walking and the maximum angle during walking (knee). |
|  | **kneeankle_dif** | The difference between the angle while standing before walking and the maximum angle during walking (knee+ankle). |
|  | **hipankle_dif** | The difference between the angle while standing before walking and the maximum angle during walking (hip joint+ankle). |
|  | **hipknee_dif** | The difference between the angle while standing before walking and the maximum angle during walking (hip joint+knee). |
|  | **all_max_dif** | The difference between the angle while standing before walking and the maximum angle during walking (hip joint+knee+ankle). |

**Supplementary Table S4.** Variables for joints used in gait analysis

|  | | **Sex** | **Age (sd)** | Height **(sd)** | **Weight (sd)** |
| --- | --- | --- | --- | --- | --- |
| **Sarcopenia** | **Female** | **15** | 77.5(9.3) | 153.0(5.6) | 50.1(7.2) |
|  | **Male** | 8 | 78.4(7.5) | 168.2(8.3) | 63.5(8.1) |
|  | **Total** | 23 | 77.8(8.6) | 158.3(9.9) | 54.8(9.8) |
|  | | **Sex** | **Age (sd)** | Height **(sd)** | **Weight (sd)** |
| **Control** | **Female** | 18 | 79.2(6.4) | 153.5(6.1) | 53.8(8.1) |
|  | **Male** | 9 | 71.2(4.6) | 166.9(5.5) | 71.71(12.0) |
|  | **Total** | 27 | 76.4(7.0) | 158.2(8.7) | 60.0(12.8) |

| **Mann-Whitney U test** | **Age** | **Total number of steps (n)** | **Cadence**  **(steps/min)** | **R_double_support**  **(%)** | **R_single_support**  **(%)** | **L_double_support**  **(%)** | **L_single_support**  **(%)** |
| --- | --- | --- | --- | --- | --- | --- | --- |
| **p-value** | 0.546 | 0.654 | 1.000 | 0.908 | 0.285 | 0.790 | 0.990 |

| **Mann-Whitney U test** | **knee_mean** | **knee_range** | | **hip_mean** | | **hip_range** | | **shoulder_angle_mean** | | **shoulder_angle_range** | | **ankle_mean** | | **ankle_range** |
| --- | --- | --- | --- | --- | --- | --- | --- | --- | --- | --- | --- | --- | --- | --- |
| **p-value** | 0.521 | 0.021^*^ | | <0.000^***^ | | 0.139 | | 0.001^**^ | | <0.000^***^ | | <0.000^***^ | | 0.161 |
| **Mann-Whitney U test** | **all_max_dif** | | **hipknee_dif** | | **hipankle_dif** | | **kneeankle_dif** | | **knee_dif** | | **ankle_dif** | | **hip_dif** | |
| **p-value** | <0.000^*^ | | 0.003^**^ | | <0.000^***^ | | <0.000^***^ | | <0.000^***^ | | <0.000^***^ | | <0.000^***^ | |

(^***^ : p-value<0.001, ^**^ : p-value<0.01, ^*^ : p-value<0.1)
